# 

**Published:** 1897-01-02

**Authors:** 


ABSOLUTELY PURE, therefore BEST,

**CADBURY'S COCOA**

"The standard of highest purity at present attainable."—*Lancet.*

**CADBURY'S COCOA**

NO ALKALIES USED.

LIBRARY
Indexed
S. G. O.

### THE HOSPITAL

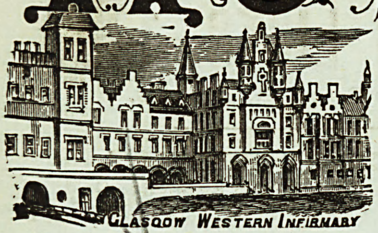

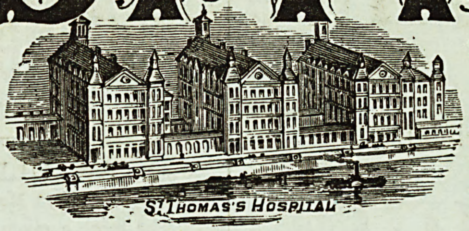

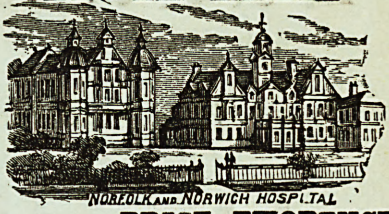

No. 536. Vol XXI.

SATURDAY, JAN 2ND, 1897.

[Registered at the General Post Office as a Newspaper for transmission in the United Kingdom and Abroad.]

